# Retraction: Structural characterization of *Momordica charantia* L. (Cucurbitaceae) oligopeptides and the detection of their capability in non-small cell lung cancer A549 cells: induction of apoptosis

**DOI:** 10.1039/d2ra90040h

**Published:** 2022-04-27

**Authors:** Laura Fisher

**Affiliations:** Royal Society of Chemistry Thomas Graham House, Science Park, Milton Road Cambridge CB4 0WF UK advances-rsc@rsc.org

## Abstract

Retraction of ‘Structural characterization of *Momordica charantia* L. (Cucurbitaceae) oligopeptides and the detection of their capability in non-small cell lung cancer A549 cells: induction of apoptosis’ by Jiao Dong *et al.*, *RSC Adv.*, 2019, **9**, 8300–8309, https://doi.org/10.1039/C9RA00090A.

The Royal Society of Chemistry hereby wholly retracts this *RSC Advances* article due to a significant amount of unattributed text overlap throughout the article, and particularly in the Results and Discussion sections, with a paper by Hongyu Li *et al.* in *Frontiers in Pharmacology* that was not cited in the article.^1^

Jiao Dong, Xianxin Zhang, Chunxiao Qu, Xuedong Rong and Jie Liu oppose the retraction. Yiqing Qu has been informed but has not responded to any correspondence regarding the retraction.

Signed: Laura Fisher, Executive Editor, *RSC Advances*

Date: 29^th^ March 2022

## Supplementary Material
